# Additive Manufacturing of Magnetostrictive Fe–Co Alloys

**DOI:** 10.3390/ma15030709

**Published:** 2022-01-18

**Authors:** Kenya Nakajima, Marc Leparoux, Hiroki Kurita, Briac Lanfant, Di Cui, Masahito Watanabe, Takenobu Sato, Fumio Narita

**Affiliations:** 1 Department of Materials Processing, Graduate School of Engineering, Tohoku University, Sendai 980-8579, Japan; kenya.nakajima.p3@dc.tohoku.ac.jp; 2 Swiss Federal Laboratories for Materials Science and Technology (Empa), CH-3602 Thun, Switzerland; Marc.Leparoux@empa.ch (M.L.); Briac.Lanfant@empa.ch (B.L.); Di.Cui@empa.ch (D.C.); 3 Department of Frontier Sciences for Advanced Environment, Graduate School of Environmental Studies, Tohoku University, Sendai 980-8579, Japan; kurita@material.tohoku.ac.jp; 4 Research and Development Department, Tohoku Steel Co., Ltd., Muratamachi 989-1393, Japan; m-watanabe@tohokusteel.com (M.W.); take_sato@tohokusteel.com (T.S.)

**Keywords:** additive manufacturing, backscattered diffraction, iron alloys, magnetostriction, magnetoelasticity, magnetic properties, microstructure

## Abstract

Fe–Co alloys are attracting attention as magnetostrictive materials for energy harvesting and sensor applications. This work investigated the magnetostriction characteristics and crystal structure of additive-manufactured Fe–Co alloys using directed energy deposition. The additive-manufactured Fe–Co parts tended to exhibit better magnetostrictive performance than the hot-rolled Fe–Co alloy. The anisotropy energy Δ*K*_1_ for the Fe–Co bulk, prepared under a power of 300 W (referred to as bulk−300 W), was larger than for the rolled sample. For the bulk−300 W sample in a particular plane, the piezomagnetic constant *d* was large, irrespective of the direction of the magnetic field. Elongated voids that formed during additive manufacturing changed the magnetostrictive behavior in a direction perpendicular to these voids. Magnetic property measurements showed that the coercivity decreased. Since sensors should be highly responsive, Fe–Co three-dimensional parts produced via additive manufacturing can be applied as force sensors.

## 1. Introduction

Magnetostriction occurs in materials under applied external magnetic fields, where spontaneous magnetization is aligned with the magnetic field direction and the elastic energy changes because of an interaction between spins, producing ferromagnetism [1]. This phenomenon means that the direction of elongation also changes because of the magnetic domains; that is, the sum of the shape changes due to magnetic domains is the magnetostriction. The extent of magnetostriction is approximately 1 ppm, which is the same magnitude as the thermal expansion coefficient of metal; thus, even small phenomena greatly influence the magnetic properties of magnetostrictive materials, and these materials are expected to have applications in, for example, sensors and actuators [2,3,4,5,6,7].

Like the magnetic anisotropy energy of a crystal, magnetostriction depends on crystal orientation [8,9,10,11]. Magnetostrictive materials are influenced by numerous factors, including the textures of polycrystalline materials, crystal orientation, and lattice strain. The magnitude of magnetostriction is usually greatest along the axis of easy magnetization (e.g., in Fe–Ga, the <100> direction), and the greatest magnetostriction can be achieved with single-crystalline or polycrystalline materials with highly aligned microstructures, rather than materials without such aligned microstructures [12,13]. From a crystallographic viewpoint, single crystals exhibit the best magnetostrictive properties; however, their mechanical properties are inferior to those of polycrystalline materials. Columnar-oriented structures obtained via rolling also exhibit strong magnetostriction [14]; however, these materials must be annealed after rolling to remove internal defects induced by work hardening [15].

Fe–Co alloys exhibit large magnetostriction (80–140 ppm), are inexpensive compared to a giant magnetostrictive Tb_x_Dy_1−x_Fe_2_ (Terfenol-D) alloy, and exhibit excellent mechanical properties compared to inexpensive Fe-based amorphous alloys. They can be processed into rods, plates, and wires and show excellent potential in magnetostriction-associated applications [16,17,18,19,20]. Compared with pure Fe, greatly enhanced magnetostriction occurs in Fe–Co alloys within the two-phase (body-centered cubic (bcc) and face-centered cubic (fcc)). However, precipitation of the fcc phase leads to diminished magnetostriction because this phase has a low magnetic moment.

Additive manufacturing technology has recently attracted attention for forming complex shapes that cannot be formed, or are extremely difficult to form, using conventional fabrication technologies [21,22,23]. Additive manufacturing can be used for near-net shaping of metals from computer-aided design data and can tailor the crystal orientation by controlling the microstructure growth conditions. Thus, additive manufacturing technology has been used to fabricate single-crystal turbine blades and implants with high mechanical biocompatibility [24,25,26].

In additive manufacturing, temperature gradients are large in the building direction, and movement of the solidification interface in this direction is dominant. Thus, crystals are easily oriented in the building direction. Several studies have reported a preferential <100> orientation for cubic crystalline metals [15,27,28,29,30,31]. In laser metal deposition, which is a type of directed energy deposition (DED), as for all solidification processes, the conditions under which epitaxial growth occurs are defined by the columnar-equiaxed transition theory. This theory has been applied to, for example, the repair of single-crystal turbine blades [31,32].

DED is an up-and-coming 3D printing technology because it enables parts to be manufactured faster than can be achieved with powder-bed manufacturing. It provides a unique possibility to feed powders of different metals simultaneously and fuse them to form functionally graded materials [33]. The additive production of sandwich structures with two different metals is also possible. In recent years, bonded two-metal layers comprising an Fe–Co layer and a Ni or SUS 304 layer have been fabricated as high-performance magnetostrictive energy harvesters [34]. They are expected to provide a higher output voltage when fabricated via DED than when fabricated via thermal diffusion bonding.

For the fabrication of Fe–Co bulk materials with complex structures, AM may be a suitable approach. Furthermore, the post-processing steps such as rolling and heat treatment generally performed after conventional fabrication should not be necessary after AM processing. 

In the present study, the magnetostriction characteristics and crystal structure of Fe–Co, bulk fabricated via DED, were investigated. Research and development related to magnetostrictive materials was carried out to date [2,4,5,6,16,17,18,19,20,34], with the novelty of this work being that the magnetostrictive alloys were fabricated via additive manufacturing technology and the magnetostrictive characteristics clarified.

## 2. Experimental Procedure

In this work, magnetostrictive Fe_30_Co_70_ alloy (Tohoku Steel. Co., Ltd., Muratamachi, Japan) was considered. Fe_30_Co_70_ alloy has better plastic processing ability and can be used to prepare complex samples using mechanical processing methods such as rolling. The results of magnetostriction (sum of the strain parallel to the magnetic field and strain perpendicular to it) for the whole spray-cast Fe_100-*x*_Co*_x_* binary series [35] showed, in fact, that with an increase in Co content, magnetostriction increased, reaching a maximum of 137 ppm at Fe_30_Co_70,_ and then dropped precipitously. The results also showed that magnetostriction reached a broad plateau at about 110 ppm for Co compositions between 40 and 60 at.%. However, it is difficult to control the direction of the easy axis of magnetization. Hence, when manufacturing involves controlling the easy axis of the pipe in the circumferential or radial direction, for example, the necessity of using a 3D printing method becomes evident.

The binary phase diagram of Fe–Co can be found in [36]. Fe_30_Co_70_ powder with an average particle size greater than 120 μm was prepared via gas atomization. However, the DED facility used in this work, Mobile 1.0 (BeAM, Strasbourg, France), requires particles smaller than 105 μm, ideally between 40 and 90 μm. Therefore, the starting powder was ball-milled under the conditions listed in Table 1.

**Figure 1:**
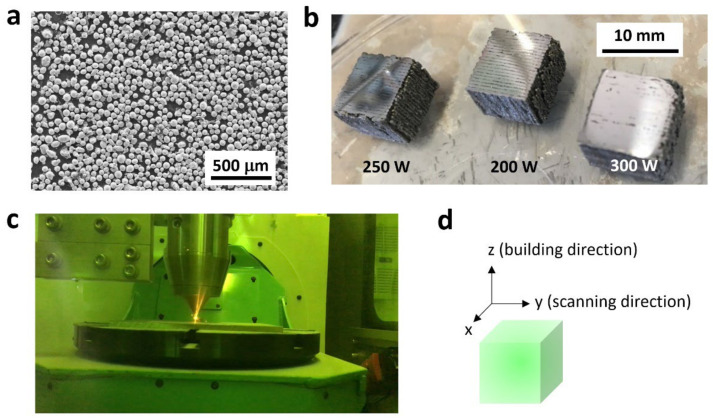
(**a**) SEM image of Fe–Co powder; (**b**) additive-manufactured Fe–Co bulk alloy samples prepared via DED; (**c**) a DED machine (BeAM mobile machine 1.0) during fabrication of the sample; (**d**) arrangement of the samples on the base plate with the scanning and building directions.

Milling was performed to reduce the particle size from ~120 to ~45 μm. Up-milling is a type of milling in which the rotation of the wheel and movement of the workpiece are in opposite directions. The resultant average particle size was 45 μm according to scanning electron microscopy (SEM) (Hitachi S-4800, Chiyoda, Japan) (Figure 1a). Fe–Co cubes with dimensions 1 × 1 × 1 cm^3^ (Figure 1b) were then fabricated on a steel plate (316L, 1 cm thick) within the DED facility (Figure 1c) under a controlled atmosphere (O_2_ < 10 ppm, H_2_O < 150 ppm). Additional information about the DED facility and process is available elsewhere [37,38]. The printing conditions were a hatch space of 0.56 mm, a scanning speed of 1000 mm/min, and a layer thickness of 0.2 mm. The laser power was set to 200, 250, or 300 W, corresponding to an energy density (calculated using Equation (A1) in Appendix A [39,40]) equal to 107.1, 133.9, or 160.7 J/mm^3^, respectively. The scanning and stacking directions were the *y*-direction and the *z*-direction, respectively (Figure 1d). A constant powder feed rate of 4 g/min was maintained for all of the fabricated structures.

The density of fabricated Fe–Co bulk samples was measured using Archimedes’ method. The crystal structure, orientation, and grain size were evaluated using SEM (SU-70, Hitachi Ltd., Tokyo, Japan) and electron backscatter diffraction (EBSD, Ametek, Berwyn, PA, USA) on the *x*–*y*, *y*–*z*, and *z*–*x* planes after ion milling (IM4000, Hitachi, Ltd., Tokyo, Japan). Figure 1b shows a photograph of the additive-manufactured Fe–Co bulk sample.

To measure the magnetic and magnetostrictive properties, each plane (*x*–*y*, *y*–*z*, *z*–*x*) of the bulk was cut out and polished to a width of approximately 6 × 6 mm^2^ and a thickness of approximately 0.2 mm. However, measuring the *x*–*y* plane of the Fe–Co bulk obtained at 200 W (hereinafter referred to as “bulk−200 W”) was difficult because this brittle bulk sample developed numerous cracks introduced by the polishing process. A hot-rolled Fe_30_Co_70_ alloy was also prepared for comparative measurements.

**Figure 2:**
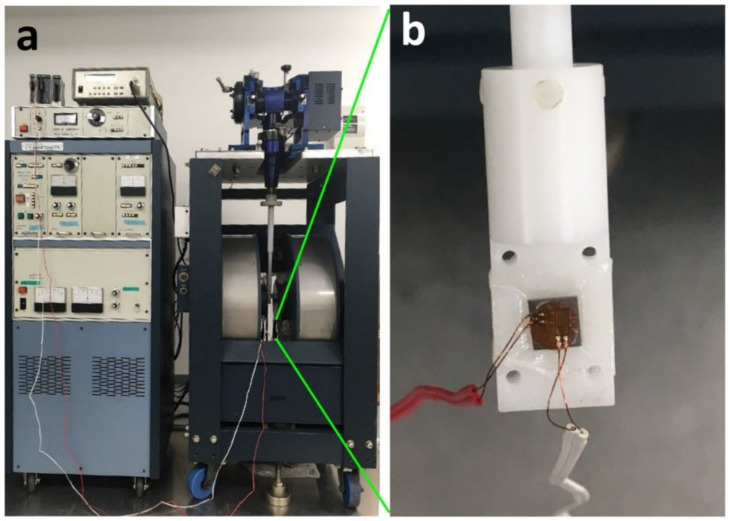
(**a**) VSM apparatus for magnetic and magnetostrictive measurements; (**b**) a two-axis strain gauge.

The magnetic properties (i.e., the saturation magnetization, remanent magnetization, and coercivity) were measured using a vibrating sample magnetometer (VSM; BHV-50H, Riken Denshi Co., Tokyo, Japan), as shown in Figure 2a. The vibration was applied perpendicular to the direction of the magnetic field. Magnetostriction was also measured with a biaxial gauge (Figure 2b, Kyowa Electronic Instruments Co., Ltd, Tokyo, Japan), using a strain-gauge method when a magnetic field was applied parallel and perpendicular to each direction (*x*–*y*, *y*–*z*, and *z*–*x* planes), as with the EBSD measurements. The magnetic anisotropy energy was then calculated using the results obtained from VSM measurements.

## 3. Results and Discussion

The properties of the Fe–Co bulk obtained at 250 W and 300 W (hereinafter referred to as “bulk−250 W” and “bulk−300 W”), as well as the hot-rolled Fe–Co sample, are discussed in this section. Table 2 lists the results of the density measurement for each bulk sample.

The theoretical density of Fe–Co is 8.58 g/cm^3^; however, the density of all additive-manufactured Fe–Co bulk alloy samples prepared via DED was lower than the theoretical density. The density of the bulk−300 W sample was the closest to the theoretical value. A high input energy density is required to attain a high relative density. Increasing the energy density requires reductions in the scanning speed, hatch spacing, and layer thickness, according to Equation (A1). Here, we considered that high porosity has an essential effect on the performance of the samples. Hence, no attempt was made to increase the energy density.

**Figure 3:**
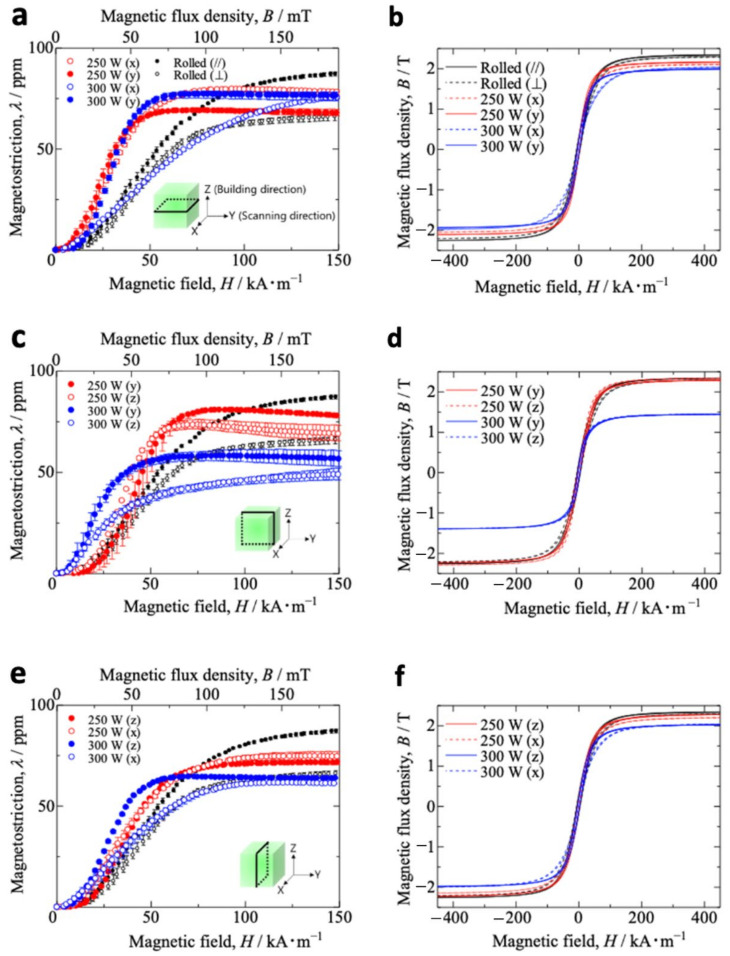
The results of magnetostriction *λ* vs. magnetic field *H* curves of additive-manufactured Fe–Co samples in the (**a**) *x*–*y* plane, (**c**) *y*–*z* plane, and (**e**) *z*–*x* plane. The results of the *B*–*H* curve of additive-manufactured Fe–Co samples in the (**b**) *x*–*y* plane, (**d**) *y*–*z* plane, and (**f**) *z*–*x* plane. The same sample was measured three times, and the mean was plotted. Error bars indicate standard deviation.

Figure 3a shows the magnetostriction *λ* vs. magnetic field *H* curves for the additive-manufactured Fe–Co samples in the *x*–*y* plane. Here, only the strain parallel to the magnetic field is considered. The black dots denote the results for the rolled samples (*λ* is approximately 80 ppm at 125 kA/m and is consistent with data for Fe_30_Co_70_ alloy reported by Han et al. [35]), and the red dots denote the results of additive-manufactured samples produced at 250 W. The initial slopes of the curves for the Fe–Co bulk−250 W (red solid and open circles) were larger than those found for the rolled samples (black solid and open circles). For a one-dimensional problem, the constitutive equations for magnetostrictive materials are given as [3]:

*ε* = *sσ* + *dʹH* (1)

*B* = *dʹσ* + *μH* (2)

where *σ* and *ε* are stress and strain, *B* and *H* are the magnetic flux density and magnetic field intensity, and *s*, *dʹ*, and *μ* are the elastic compliance, magnetoelastic constant, and magnetic permeability, respectively. The magnetoelastic constant is given by

*dʹ* = *d* + *mH* (3)

where *d* is the piezomagnetic constant and *m* is the second order magnetoelastic constant [41,42]. The slope of the magnetostriction vs. magnetic field curve represents the piezomagnetic constant *d*, which is a parameter directly related to the performance of magnetostrictive devices. Thus, the additive-manufactured Fe–Co samples can perform better as magnetostrictive materials than traditional rolled Fe–Co samples. The results also show that the initial slope of the curve corresponding to Fe–Co bulk−250 W under the *y*-direction magnetic field was larger than that under the *x*-direction magnetic field. The Fe–Co bulk−300 W (blue solid and open circles) results indicate that increasing the power reduced the initial slope of the curve, irrespective of the applied magnetic field direction. For the Fe–Co bulk−250 W, the magnitude of the magnetic field at which the magnetostriction reached saturation was much smaller than the rolled sample. A similar tendency was observed for the Fe–Co bulk−300 W under the *y*-direction magnetic field. However, for the Fe–Co bulk−300 W under the *x*-direction magnetic field, magnetostriction increased linearly with increasing magnetic field strength and did not become saturated below 150 kA/m. Notably, the magnetostriction for the Fe–Co bulk−250 W under the *y*-direction magnetic field was lower than that for the Fe–Co bulk−250 W under the *x*-direction magnetic field and the Fe–Co bulk−300 W under *x*- and *y*-direction magnetic fields. Figure 3c shows similar results for the *y*–*z* plane. Interestingly, for the *y*–*z* plane, the initial slopes of the Fe–Co bulk−300 W curves were much larger than those of the Fe–Co bulk−250 W and rolled samples. Thus, increasing power increased the slope of the curves. However, increasing power decreased the magnetostriction. Figure 3e shows similar results for the *z*–*x* plane. Similar to the Fe–Co bulk−300 W under the *x*-direction magnetic field for the *x*–*y* plane, the magnetostriction of the Fe–Co bulk−300 W under the *x*-direction magnetic field increased linearly with increasing magnetic field strength. The magnetostriction then gradually reached saturation. Figure 3b,d,f show the *B*–*H* curves for the *x*–*y* plane, *y*–*z* plane, and *z*–*x* plane, respectively. Generally, the magnetic polarization increases with increasing density. It seems that the magnetic properties (e.g., the magnetostriction or the magnetic flux density) decrease with increasing laser power. It is expected that the voids affect these magnetic properties.

**Figure 4:**
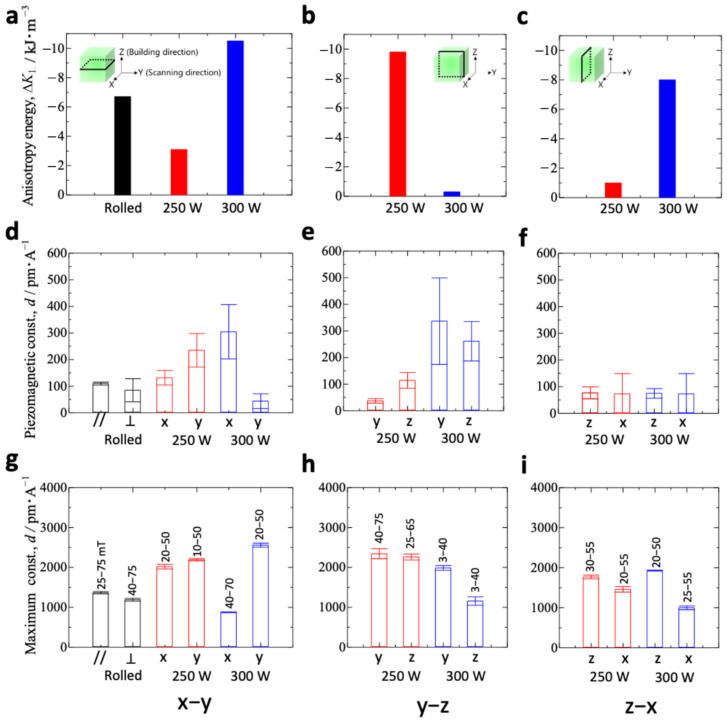
The anisotropy energy Δ*K*_1_ for additive-manufactured Fe–Co bulk alloy samples on the (**a**) *x*–*y* plane, (**b**) *y*–*z* plane, and (**c**) *z*–*x* plane. The results of the piezomagnetic constant *d* for the (**d**) *x*–*y* plane, (**e**) *y*–*z* plane, and (**f**) *z*–*x* plane. The maximum piezomagnetic constant *d* for (**g**) the *x*–*y* plane, (**h**) *y*–*z* plane, and (**i**) *z*–*x* plane. The same sample was measured three times and the mean plotted. Error bars indicate standard deviation.

Figure 4a shows the anisotropy energy Δ*K*_1_ for additive-manufactured Fe–Co samples in the *x*–*y* plane. The results for the rolled sample are also shown to aid comparison. Details of the calculation of Δ*K*_1_ are provided in Appendix B [43]. The anisotropy energy Δ*K*_1_ for the Fe–Co bulk−300 W was larger than for the rolled sample. Figure 4b,c show the results for the additive-manufactured Fe–Co samples in the *y*–*z* plane and *z*–*x* plane, respectively. In contrast to the anisotropy energy Δ*K*_1_ in the *x*–*y* plane, the *y*–*z* plane for the Fe–Co bulk−300 W was smaller than that for the rolled sample. Figure 4d–f show the piezomagnetic constant *d* for the *x*–*y* plane, *y*–*z* plane, and *z*–*x* plane, respectively. These constants were obtained from the initial slope of the curves in Figure 3a,c,e, respectively. The results for the rolled sample are also shown in Figure 4d. The piezomagnetic constant *d* for the rolled sample under the rolling-direction magnetic field was approximately 110 pm/A. However, it was 80 pm/A in the magnetic field vertical to the rolling direction. On the *x*–*y* plane, the piezomagnetic constant *d* for the Fe–Co bulk−300 W under the *x*-direction magnetic field was the largest (approximately 300 pm/A), whereas the *d* for the Fe–Co bulk−300 W under the *y*-direction magnetic field was the smallest (~40 pm/A). This result is attributed to the high anisotropy energy. In the *y*–*z* plane, the values of *d* for the Fe–Co bulk−300 W under the *y* and *z*-directions are equally large (340 and 260 pm/A, respectively). This result is attributed to the small anisotropy energy. Interestingly, the Fe–Co bulk−300 W shows anisotropic magnetostriction in the *x*–*y* plane and isotropic magnetostriction in the *y*–*z* plane. Figure 4g–i show the maximum piezomagnetic constant *d* corresponding to Figure 4d–f, respectively, obtained from the maximum slope of the curves in Figure 3a,c,e. The values in the graph are the values of the magnetic flux density where the slope is maximal. The maximum piezomagnetic constant *d* for the additive-manufactured Fe–Co samples was larger than for the rolled sample. Note that the magnetic flux density value, where *d* indicates the maximum, is small in the *y*–*z* plane for the Fe–Co bulk−300 W under the *y*- and *z*-directions.

**Figure 5:**
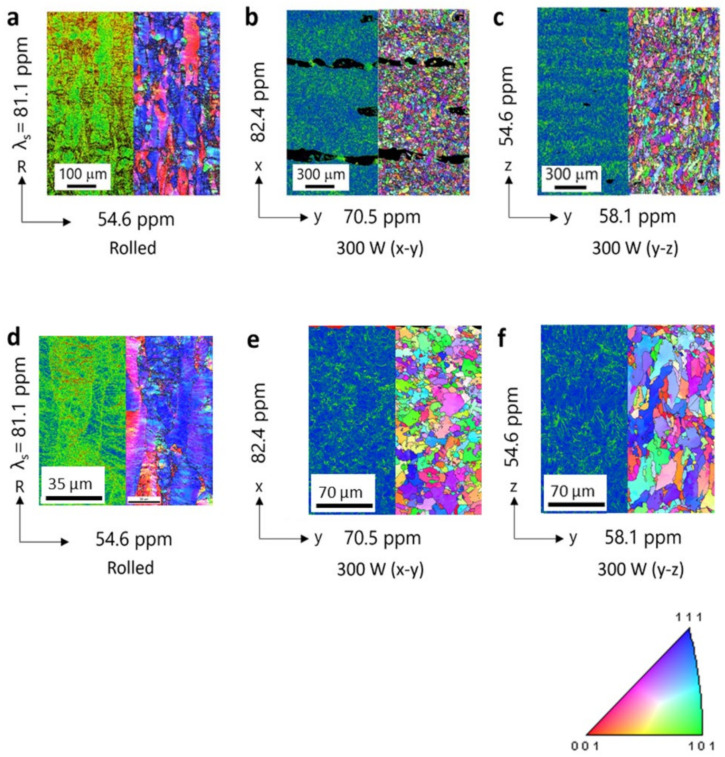
Microstructure features obtained via EBSD analysis; each figure shows the KAM map and IPF map for (**a**) rolled, (**b**) bulk−300 W (*x*–*y* plane), and (**c**) bulk−300 W (*y*–*z* plane) samples; high-resolution KAM map and IPF map for (**d**) rolled, (**e**) bulk−300 W (*x*–*y* plane), and (**f**) bulk−300 W (*y*–*z* plane) samples.

Figure 5a shows the kernel average misorientation (KAM) map and inverse pole figure (IPF) map obtained from EBSD analysis of the rolled sample. The magnetostrictions *λ*_s_ = (2/3) × (*λ_//_* − *λ*_⊥_) parallel and normal to the rolling direction are also shown. The microstructure was elongated in the rolling direction, and magnetostriction was larger in the plane parallel to this preferential orientation. Figure 5b,c shows similar results for the bulk−300 W samples in the *x*–*y* plane and *y*–*z* plane, respectively. A high degree of crystal orientation was not observed for the bulk−300 W samples in the *x*–*y* plane. The KAM and IPF maps show that voids were preferentially aligned in the *y*-direction (scanning direction) because of a large hatch space between two subsequent layers at the interface. The deformation under the magnetic field in the *x*-direction must have been small, as indicated by the presence of voids in the *y*-direction; thus, the piezomagnetic constant *d* under the magnetic field in the *x*-direction (Figure 4d) was larger than in the *y*-direction. These voids appear to contribute to magnetostriction in the *x*-direction. The extent of magnetostriction in the *x*-direction was similar to that in the rolled sample. The KAM map indicated strong distortion near the grain boundaries for the bulk−300 W samples in the *y*–*z* plane. This distortion in the *y*-direction contributed to an increase in the piezomagnetic constant *d* under the *y*-direction magnetic field (Figure 4e) and an increase in magnetostriction in the *y*-direction. The IPF map also confirms that a columnar crystal structure grew in the *z*-direction (building direction).

On the other hand, in the case of magnetostrictive Fe-Co alloys, Mössbauer spectrometry appears to represent a very effective tool for conducting phase purity analysis [44]. This challenging task will be addressed in due course.

Table 3 summarizes the results of magnetic measurements for the rolled and AM bulk−300 W samples. The bulk−300 W samples had a lower saturation magnetization than the rolled sample. The reduced saturation magnetization of the additively manufactured samples, when compared to the rolled sample, can be explained, in part, by the lower density of the material. The saturation magnetization in the *y*–*z* plane is especially low. Furthermore, the bulk−300 W samples exhibited lower remanent magnetization and lower coercivity than the rolled sample. It seems that the grain refinement effect, as shown in Figure 5, works effectively to reduce coercivity [45]. These results indicate that these samples could be used for low-magnetic-field sensors because of their lower coercivity. Fortunately, the piezomagnetic constant *d* defined in the low-magnetic-field region was very high for the Fe–Co bulk−300 W (see Figure 4e). Furthermore, a small remanent magnetization seemed to lead to a large piezomagnetic constant *d* (see blue bar in Figure 4d–f).

## 4. Conclusions

Fe–Co samples were prepared via AM using a DED system with various energy densities, and their magnetostrictive and magnetic properties were investigated. The magnetostrictive characteristics depended on the surface orientation respective to the building strategy. Moreover, voids that formed during AM changed the magnetostrictive properties of the additive-manufactured Fe–Co samples. For the bulk−300 W sample in the *y*–*z* plane, the piezomagnetic constant *d* was large, irrespective of the direction of the magnetic field, and was approximately 340 pm/A. This *d* value was more than three times greater than that of the rolled sample. For the bulk−300 W sample in the *x*–*y* plane, the piezomagnetic constant *d* under the *x*-direction magnetic field was also more than three times larger than that of the rolled sample, and the magnetostriction was approximately the same as in the rolled sample. Since the piezomagnetic performance increased according to the property measurements, additive-manufactured Fe–Co samples could be used in, for example, sensors with complex shapes. The properties would be further improved if the structure of the additive-manufactured samples could be controlled in one direction, similar to a single crystal. Furthermore, further clarification of the relationship between magnetostriction and porosity is needed to develop a better sensor.

## Figures and Tables

**Table 1:** The milling conditions of Fe–Co powder.

Ball size	⌀10 mm
Ball weight before milling	1200 g
Weight of first powder	135 g
Process control agent (PCA)	135%
Milling gas	Argon 4.8 (99.998%)
Milling cycles	9
Speed	350 rpm
Interval	10 min
Break	10 min
Direction of rotation	Up-milling
Milling process time	3 h

**Table 2:** Density of Fe_30_Co_70_ alloy. The calculated theoretical density is 8.58 g/cm^3^.

Laser Power (W)	Energy Density (J/mm^3^)	Density (g/cm^3^)	Error (%)
200	107.1	7.60	10.9
250	133.9	7.72	9.5
300	160.7	7.85	8.0

**Table 3:** Summary of magnetic measurements.

Sample	Plane	Direction	Saturation Magnetization (T)	Remanent Magnetization (T)	Coercivity (kA/m)
Rolled	-	R_//_	2.26	0.20	5.40
		R_⊥_	2.30	0.22	5.07
300 W	*x*–*y*	*x*	2.02	0.12	3.32
		*y*	1.97	0.15	3.12
	*y*–*z*	*y*	1.42	0.11	3.09
		*z*	1.42	0.13	3.08
	*z*–*x*	*z*	2.01	0.13	2.96
		*x*	2.02	0.12	3.15

**Figure A1:**
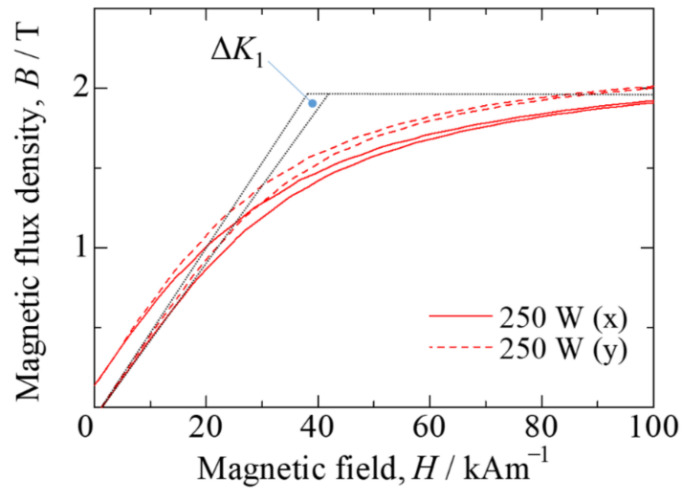
Approximately calculated anisotropy energy.

## Data Availability

Any data is not available.

## Appendix A

An additive-manufactured material has a lower porosity than a sintered compact fabricated using the conventional powder metallurgy process. In the sintering process, the voids between the powder particles become closed pores and tend to remain in the prepared sample. By contrast, in additive manufacturing, the powder particles are completely melted and easily filled. However, in the case of powder-bed fusion technology, the porosity is higher than in the sintered material if the molding conditions are unsuitable, such as a low laser power and a large hatch space. The energy density per unit volume (laser power/scanning speed/hatch space/layer thickness) is used as an index of appropriate conditions. The equation for energy density is:

(A1) $$E=\frac{P}{vth}$$

where *E* is the energy density per unit volume, *P* is the laser power, *v* is the scan speed, *t* is the layer thickness, and *h* is the hatch space.

The energy density is actually an index of the densification conditions. However, a defect, the so-called balling phenomenon, occurs via the integration of the melt and the formation of large grains when a lower laser power is irradiated at a smaller hatch space, even at the same energy density.

## Appendix B

Figure A1 shows an enlarged *B–H* curve for the Fe–Co bulk−250 W in the *x*–*y* plane. The value of anisotropy energy Δ*K*_1_ can be estimated as Δ*K*_1_ = −1/2 Δ*HB*_s_ (area indicated by the dotted black lines), where *B*_s_ is the saturated magnetic flux density. Since the initial magnetization curve could not be obtained in the present study, the slope representing the coercive force was taken as the initial slope. Furthermore, to obtain Δ*K*_1_, it was assumed that the saturated magnetic flux density had the same value irrespective of the applied magnetic field direction and that the average value of the saturated magnetic flux density in the applied magnetic field direction on each plane was used.
